# *Streptococcus pneumoniae* meningitis complicated by an intramedullary abscess: a case report and review of the literature

**DOI:** 10.1186/s13256-016-1080-7

**Published:** 2016-10-19

**Authors:** Dat T. Vo, George F. Cravens, Robert E. Germann

**Affiliations:** 1 Department of Pediatrics, John Peter Smith Hospital, 1500 South Main Street, Fort Worth, TX 76104 USA; 2 Department of Neurosurgery, John Peter Smith Hospital, 1500 South Main Street, Fort Worth, TX 76104 USA; 3 The Center for Neurological Disorders, 1010 Houston Street, Fort Worth, TX 76102 USA; 4 Department of Radiation Oncology, The University of Texas Southwestern Medical Center, 5801 Forest Park Road, Dallas, TX 75390 USA

**Keywords:** Intramedullary abscess, Meningitis, Infected syrinx, Laminectomy, Myelotomy, Case report

## Abstract

**Background:**

Intramedullary abscess is a rare neurosurgical condition that usually arises in the setting of penetrating trauma to the spinal cord, infected congenital dural sinuses, or tuberculosis.

**Case presentation:**

We describe a case of a 35-year-old African American male who presented with sepsis and a clinical picture of meningitis. The patient continued to have declining neurological status with decreasing sensation and worsening motor strength in all four extremities. He was found to have an intramedullary abscess in the cervical spinal cord that was treated with a decompressive posterior cervical laminectomy and drainage. The patient began to have a partial recovery of neurological function postoperatively. We also review the literature on intramedullary abscess that suggests the clinical presentation of our patient was a rare complication of acute meningitis.

**Conclusions:**

Intramedullary abscess formation is a rare entity, and a high index of suspicion for intramedullary abscess is the key for making the diagnosis and expediting treatment for these patients.

## Background

Meningitis complicated by an intramedullary abscess is a very rare clinical presentation first described in 1936 [1]. We present a case of a 35-year-old man who presented to our hospital with acute meningitis and was found to have a cervical intramedullary abscess, which was treated with intravenous steroids, antibiotics, and operative intervention. We also present a literature review of this rare condition.

## Case presentation

### Preadmission information

A 35-year-old African American man with a past medical history of sickle cell disease presented to our hospital by transfer from an outside hospital. Clinicians at the outside hospital originally saw him when he had a 2-day history of fever with a maximum temperature of 103 °F. The patient also complained of worsening headache, neck pain, and shortness of breath. He was admitted for suspicion of sepsis. During the evaluation process, the clinical condition of the patient worsened with increasing shortness of breath, intensifying headaches, and increasing neck pain. The patient received a lumbar puncture, the results of which were indicative of meningitis. The patient underwent magnetic resonance imaging, which revealed a possible Arnold-Chiari type I malformation and a cervical cord syrinx with associated myelitis. The patient was transferred to our hospital, and, upon arrival, he was paralyzed from approximately the level of C4 to the feet.

### History and physical examination

The patient has a history of sickle cell disease, pulmonary hypertension, and right ventricular enlargement, likely secondary to the sickle cell disease. He previously had a transthoracic echocardiogram that did not show any intracardiac shunting but did show a left ventricular ejection fraction of 50–55 %. He was taking a beta-blocker and an angiotensin-converting enzyme inhibitor for blood pressure control. He did not have any other significant history. On arrival to our hospital, the patient appeared toxic and in severe distress. The patient had moderately altered mental status but was able to follow conversation. He complained of worsening diplopia and decreasing vision from his right eye. His physical examination was significant for abnormal sensation below the level of C4 and decreased motor strength in all four extremities.

### Laboratory studies

The patient was found to have elevated blood urea nitrogen and creatinine levels, which increased from 0.9 to 4.0 mg/dL over a couple of days, likely due to acute kidney failure. The patient also had a decreased potassium level of 2.9. The patient was found to be anemic, with a hemoglobin of 6.1 g/dL, likely due to his underlying sickle cell anemia, and received a transfusion of 3 units of packed red blood cells. The patient had an elevated white blood cell count of 30.0 x 10^3^/µL. Arterial blood gas analysis revealed a pH of 7.23, partial pressure of oxygen of 115 mm Hg, bicarbonate level of 16.8 mEq/L, and base excess of −9.5 mEq/liter.

### Imaging studies

**Fig. 1:**
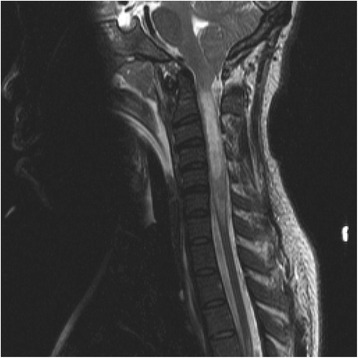
Magnetic resonance imaging scan of the cervical spinal cord of the intramedullary abscess upon admission. This sagittal T2-weighted image shows an expansile intramedullary area of increased T2 signaling present in the spinal cord from C2 to C5

The patient’s single-view chest x-ray showed bilateral lung interstitial edema, left lung pulmonary parenchymal opacity, and left pleural effusion. A magnetic resonance imaging study of the cervical spine showed fluid collection from C2 through C5 that was indicative of an intramedullary cord abscess, with edema extending to the level of C7 (Fig. 1). Magnetic resonance imaging studies of the thoracic and lumbar spine were unremarkable. A magnetic resonance imaging study of the brain showed right frontal lobe subacute infarcts, most likely within the right anterior cerebral artery territory, likely due to the underlying condition of sickle cell disease, and mild caudal herniation of the cerebellar tonsils was observed. A computed tomographic study of the thorax showed bilateral lower lobe areas of atelectasis or consolidation, suggestive of pneumonia.

### Initial treatment and management

Upon the patient’s arrival at our hospital, the concern was for worsening mentation, weakness, and sepsis, secondary to *Streptococcus* meningitis with an intramedullary abscess from C2 through C5. The patient was placed on high-dose intravenous steroids to reduce spinal cord swelling. He was placed on azithromycin, ceftriaxone, and vancomycin for broad antibiotic coverage. Concerns for pneumonia versus influenza arose because of findings in the chest x-ray obtained upon admission, and the patient was placed on oseltamivir. An arterial line, a minimally invasive hemodynamic monitor, a central venous catheter, an endotracheal tube, and a mixed venous oxygen saturation monitor were placed. Daily procalcitonin and C-reactive protein levels were obtained to monitor for treatment response. Blood and cerebrospinal fluid cultures were obtained. The patient was placed on a bicarbonate drip for metabolic acidosis but continued to have declining renal function. The patient was subsequently placed on continuous renal replacement therapy.

### Operative intervention and surgery

Two days after admission, the patient underwent surgery to treat his Arnold-Chiari type I malformation and drain his intramedullary abscess. During treatment, he underwent posterior cervical laminectomy from C2 to C6-C7, myelotomy with microscope and CO_2_, drainage of the intramedullary abscess, duraplasty of the Arnold-Chiari type I malformation and cervical cord, drain placement, and intraoperative somatosensory evoked potentials and free-run electromyography. A midline incision was made from the spinous process of C1-C2 down to C7. The lamina from C2 to C7 was then subsequently removed. The dura was opened, and the exposed cord was observed to be extremely vascularized, with the cerebrospinal fluid appearing infected. A midline myelotomy was performed from C4 to C6, opening the syrinx, and the purulent material was evacuated with significant irrigation. Then the dura and the dural canals were closed, and a drain was placed.

### Postoperative course

**Fig. 2:**
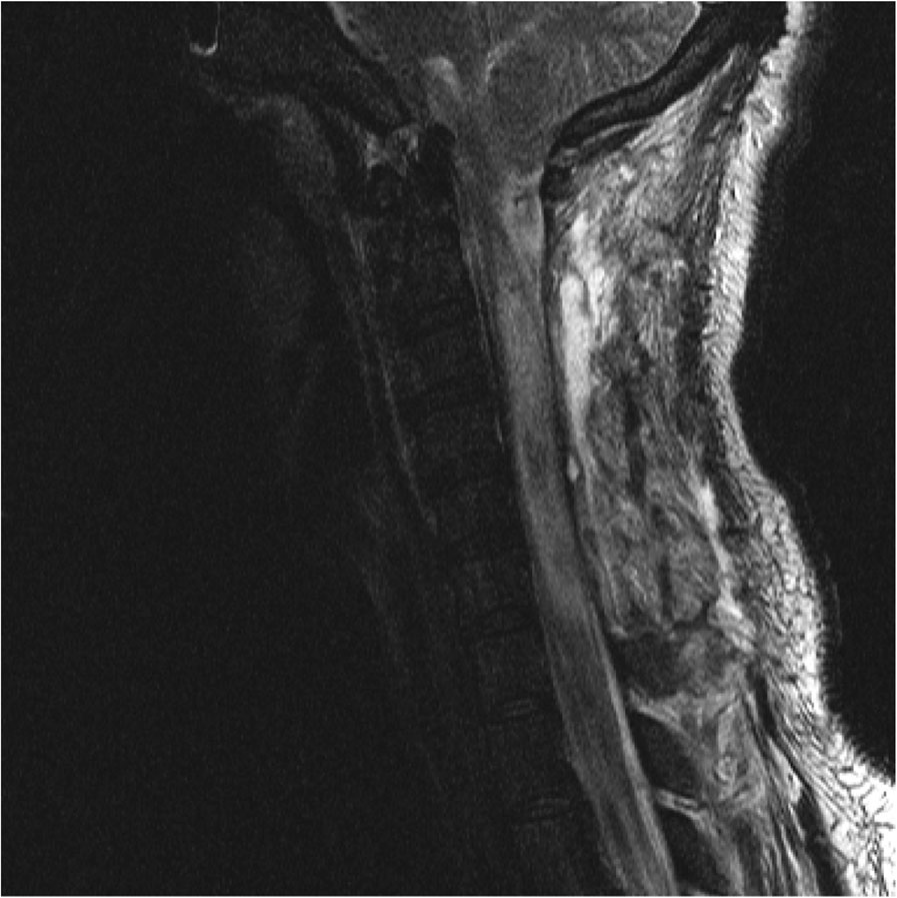
Magnetic resonance imaging scan of the cervical spinal cord of the intramedullary abscess obtained 9 days postoperatively. This sagittal T2-weighted image shows stable postoperative changes after a myelotomy and laminectomy, as well as a persistent but stable T2-weighted hyperintensity starting from the level of C2 and extending down to the level of T1

The patient was placed on cervical spine precautions. His blood cultures that were obtained from the outside hospital were found to have *Streptococcus pneumoniae* that was sensitive for ceftriaxone, which was empirically continued for a total duration of 1 month. Cultures from the syrinx and blood cultures obtained on admission did not have any growth. The patient was continued on intravenous antibiotics and steroids. He started to have improving mental status, sensation, and motor function. He underwent postoperative magnetic resonance imaging of the cervical spine 1 day after surgery, and the scans showed postsurgical drainage of the syrinx and laminectomy of C2-C6. Another magnetic resonance imaging study, obtained on postoperative day 8 (Fig. 2), showed slight improvement from the first postoperative study. The patient was extubated on postoperative day 5. At the end of his hospital stay, the patient did not have any signs or symptoms suggestive of sepsis. He had a decline in his white blood cell count as well as his procalcitonin level. He continued to experience decreased neurological function, with no sensation below the level of L2 and paresis of the bilateral lower extremities and left upper extremity. He had a nearly complete recovery of renal function after a trial of continuous renal replacement therapy. He was discharged to an inpatient spinal cord injury rehabilitation facility on postoperative day 12.

## Discussion

Intramedullary abscess is a rare neurological condition [1]. Normal spinal cord tissue has an exceptional ability to resist infection. Usually, intramedullary abscesses occur when the patient has a specific underlying condition. After an extensive literature search and review, we can summarize these conditions in four different categories: bacterial and fungal infection, penetrating trauma to the spinal cord, congenital dural sinuses, or chronic tuberculosis. Even more rarely, intramedullary abscess formation has been associated with acute bacterial meningitis.

### Bacterial and fungal infection

Despite the high level of resistance to infection by normal spinal cord tissue, authors of a few case reports have described an antecedent systemic bacterial or fungal infection with the formation of an intramedullary abscess. In one reported case, a patient presented with infective endocarditis of the mitral valve, but the patient had a previous history of radiotherapy, with the treatment field including the spinal cord, for the treatment of Hodgkin’s lymphoma [2]. This suggests that residual tissue damage caused by radiotherapy may have predisposed the patient to the formation of the intramedullary abscess. In another case, a previously healthy patient presented with infective endocarditis of the aortic valve and was later found to have an 8 × 15-mm intramedullary abscess in the cervical spinal cord [3].

Epidural abscesses are common among patients with a history of intravenous drug use, but rarely do they present with intramedullary cord abscess. In one case, a patient developed tetraplegia and was found to have an intramedullary abscess caused by *Pseudomonas cepacia* [4]. In another case, an intravenous drug user presented with a similar clinical presentation, but the abscess was infected with *Staphylococcus aureus* [5]. In a couple of other reports, one patient developed an intramedullary abscess, likely from his right to left cardiac shunt, through bypassing the normal host defenses in the lungs [6], and two separate patients had a patent foramen ovale [7, 8].

Many other microorganisms have been implicated in the formation of intramedullary abscesses, such as *Candida albicans* [9], *Nocardia asteroides* [10], *Listeria monocytogenes* [11, 12], *Cryptococcus neoformans* [13], *Brucella* [14], *Scedosporium apiospermum* [15], *Histoplasma capsulatum* [16], group F *Streptococcus* [17], *Bacteroides disiens* [18], *Streptococcus melleri* [19], and *Aspergillus fumigatus* [20]. Many of these patients have an underlying immunocompromised predisposition, such as human immunodeficiency virus (HIV) infection or agammaglobulinemia. While most cultures of intramedullary abscesses were sterile using typical laboratory testing, it has been estimated that 17 % of intramedullary abscesses are caused by the *Streptococcus* genus of organisms [21], the causative organism in our patient.

### Tuberculosis infection

Many case reports have described the formation of intramedullary abscess in patients with active tuberculosis infection or *Mycobacterium tuberculosis* in the purulent fluid in the abscess [22–30]. Most of these patients had a good recovery after surgery and the initiation of antituberculosis medications. Radiographically, the formation of a T2-weighted hyperintensity on magnetic resonance imaging studies, called the *precipitation sign*, can indicate a *Mycobacterium tuberculosis*-infected intramedullary abscess [31].

### Penetrating trauma to the spinal cord

The authors of the first description of intramedullary abscess formation reported that it occurred after spinal anesthesia following a penetrating trauma to the spinal cord [32]. Other authors have reported cases of penetrating injuries that were coincidental with intramedullary abscess formation after a stab wound [33], spinal puncture [34, 35], and transpharyngeal stab injury [36], with the most likely mechanism of infection of direct introduction microorganisms into the spine.

### Congenital dermal sinuses

Congenital dermal sinuses are multilayered tissues that can be found anywhere in the midline between the nasal bone and tailbone. Many cases of intramedullary abscesses have been described in pediatric patients with dermal sinuses that have an intramedullary component [19, 37–47]. A review of dermal sinuses and intramedullary abscess formation has been published elsewhere [48]. The authors of one case report did describe a patient with an epidermoid cyst without a dermal sinus component who eventually developed an intramedullary abscess [49].

## Conclusions

Very few cases of intramedullary abscesses occur in healthy individuals [28, 50, 51]. Many of our cited examples were patients with specific underlying conditions that perhaps predisposed them to intramedullary abscess formation. Our patient had an underlying history of sickle cell disease, and we hypothesize that his underlying condition may have caused microvascular occlusive disease resulting in microinjury in the cord and allowing for the seeding and formation of the intramedullary abscess. A similar condition of vasoocclusion was described in a patient with spinal artery occlusion, likely arising from a bacterial embolic source, who then had an acute formation of an intramedullary abscess [52].

Considering our patient’s presentation, it is also not known if he developed the intramedullary abscess before or after the onset of meningitis. A few cases have been reported that showed concurrent meningitis and intramedullary abscess formation [18, 53–55]. The authors of one report described the concurrent presentation of cervical spinal cord abscess, brain abscess, and meningitis [18]. One patient, 7 years old, had recurrent meningitis, which was a manifestation of a chronic intramedullary abscess that likely continuously seeded the intrathecal space [53]. A similar mechanism was seen in a 63-year-old patient [54]. The authors of one paper did report the development of acute meningitis after a rupture of a chronic intramedullary abscess [55].

Our patient did not have a history of chronic or recurrent meningitis, and it is likely that this was a first manifestation of both meningitis and intramedullary abscess for this patient. However, as described above, most patients who develop an intramedullary abscess have some predisposition to the condition. In the case of our patient, it is likely that his sickle cell condition, which can cause vasoocclusive disease and/or an immunocompromised state, drove the pathogenesis toward intramedullary abscess formation. Unfortunately, the status of the sickle cell disease in our patient was unknown, given the clinical circumstances and urgency surrounding his case. The patient would be likely to have a functional asplenia, starting early in childhood, which may predispose one to serious infections. Additionally, echocardiography was not performed to rule out pneumococcal endocarditis, which may occur in a triad of symptoms called Austrian syndrome [56]. However, the use of broad-spectrum antibiotics may have reduced the risk of pneumococcal endocarditis, as discussed in other reports [57]. While intramedullary abscess formation is a rare entity, we propose that a high index of suspicion for intramedullary abscess is the key to the diagnosis and expedited treatment for these patients.

## Abbreviations

ACE: Angiotensin-converting enzyme
CO_2_: Carbon dioxide
